# Potential Nephroprotective Effect of Kaempferol: Biosynthesis, Mechanisms of Action, and Clinical Prospects

**DOI:** 10.1155/2024/8907717

**Published:** 2024-09-30

**Authors:** Maulana Yusuf Alkandahri, Asman Sadino, Barolym Tri Pamungkas, Zulpakor Oktoba, Maya Arfania, Nia Yuniarsih, Eko Sri Wahyuningsih, Yuliani Dewi, Sri Ayu Winarti, Sri Tantia Dinita

**Affiliations:** ^1^ Department of Pharmacology and Clinical Pharmacy Faculty of Pharmacy Universitas Buana Perjuangan Karawang, Karawang, West Java, Indonesia; ^2^ Department of Pharmacy Faculty of Mathematics and Natural Science Universitas Garut, Garut, West Java, Indonesia; ^3^ Department of Pharmaceutical Biology Faculty of Pharmacy Universitas Mulawarman, Samarinda, East Kalimantan, Indonesia; ^4^ Department of Pharmacy Faculty of Medicine Universitas Lampung, Bandar Lampung, Indonesia; ^5^ Department of Pharmaceutical Technology Faculty of Pharmacy Universitas Buana Perjuangan Karawang, Karawang, West Java, Indonesia; ^6^ Department of Pharmaceutical Biology Faculty of Pharmacy Universitas Buana Perjuangan Karawang, Karawang, West Java, Indonesia; ^7^ Faculty of Pharmacy Universitas Buana Perjuangan Karawang, Karawang, West Java, Indonesia

## Abstract

Kidney is an essential organ that is highly susceptible to cellular injury caused by various toxic substances in the blood. Several studies have shown that untreated injuries to this organ can cause glomerulosclerosis, tubulointerstitial fibrosis, and tubular cell apoptosis, leading to kidney failure. Despite significant advancements in modern treatment, there is no fully effective drug for repairing its function, providing complete protection, and assisting in cell regeneration. Furthermore, some available medications have been reported to exacerbate injuries, showing the need to explore alternative treatments. Natural drugs are currently being explored as a new therapeutic strategy for managing kidney diseases. Kaempferol, a polyphenol found in plants, including vegetables, legumes, and fruits, has been extensively studied in various nephrotoxicity protocols. The compound has been reported to have potential as a nephroprotective agent with beneficial effects on various physiological pathways, such as CPL-induced kidney injury, DOX, LPO, ROS, RCC, and diabetic nephropathy. Therefore, this study aims to provide a brief overview of the current nephroprotective effects of kaempferol, as well as its molecular mechanisms of action, biosynthesis pathways, and clinical prospects.

## 1. Introduction

Kidney is an essential organ with several physiological functions, including maintaining fluid homeostasis by filtering and excreting metabolites and minerals from the blood. In addition, it plays an important role in eliminating nitrogen waste through urine, contributing to glucose metabolism, erythropoiesis, blood pressure regulation, hormones, and enzyme production [1, 2]. A previous report showed that it filtered approximately one hundred and eighty litres of blood daily, equivalent to 4 times the amount passing through other organs. This situation increases the exposure to toxins in the blood, leading to increased susceptibility to kidney damage and diseases [3, 4]. Pathways leading to this damage are classified into prerenal, intrinsic, and postrenal factors based on causation [5]. Prerenal diseases are typically associated with decreased kidney perfusion or systemic circulation changes, disrupting the glomerular filtration rate (GFR) and causing more severe structural changes. These alterations can be observed through clinical analyses, such as increased serum creatinine, blood urea nitrogen (BUN), and urine flow fluctuations [6, 7]. Based on previous reports, prerenal damage factors comprise a spectrum of diseases, including rhabdomyolysis, hypertension, liver damage, systemic infections, autoimmune disorder, diabetes mellitus, shock, bleeding, intravascular volume depletion, trauma, and gut microbiota disorders [8]. Meanwhile, intrinsic factors leading to direct injury include kidney cancer, systemic lupus erythematosus (SLE), atheroembolic kidney disease, proteinuria, drug toxicity, trauma, arthritis, congenital abnormalities, Wegener's granulomatosis, heavy metal exposure, and proteinuria. Common diagnoses caused by intrinsic damage include glomerulonephritis, nephrotoxic acute tubular necrosis, and ischemic acute tubular necrosis [9]. Postrenal diseases have also been reported to contribute to kidney failure and are associated with urinary flow disorders, such as urinary tract infection (UTI), lithiasis, ureter and urethra obstruction due to blood clotting, and tumor growth causing increased pressure in the tubules, thereby disrupting the GFR [10]. Studies suggest that addressing kidney diseases with pharmacological treatment at various stages, considering their main causes, can prevent progression to end-stage renal failure. However, most drugs used can cause even more severe damage to the organ, showing the need to develop alternative treatments with minimal side effects [5].

Medicinal plants have traditionally been extensively used as therapies for diseases affecting various organs, including the kidney [11]. Nephroprotective agents from herbal plants have been reported to have the ability to reduce kidney damage processes, such as intraglomerular hemodynamic changes, tubular necrosis, interstitial nephritis, and glomerulonephritis [12]. Approximately 80% of patients worldwide have used these medicines due to their widespread availability, low toxicity, pharmacological activity, chemical diversity, and lower side effects compared to synthetic drugs [13]. An essential nephroprotective agent derived from herbal plants is kaempferol (tetrahydroxyflavone). This compound is one of the most common flavonoid aglycones found in glycoside form, with four hydroxyl groups located at positions 3, 5, 7, and 4′ [14]. Several studies have shown that it is often found in various parts of plants and plant-based foods, such as strawberries, legumes, black tea, spinach, and green onions [15, 16]. Kaempferol and its derivatives have also been proven to possess nephroprotective activities through various mechanisms [4]. Therefore, this study aims to discuss the effects of kaempferol for the prevention and treatment of kidney diseases and to assess its biosynthesis pathways, molecular action mechanisms, and clinical prospects.

## 2. Biosynthesis of Kaempferol

**Figure 1:**
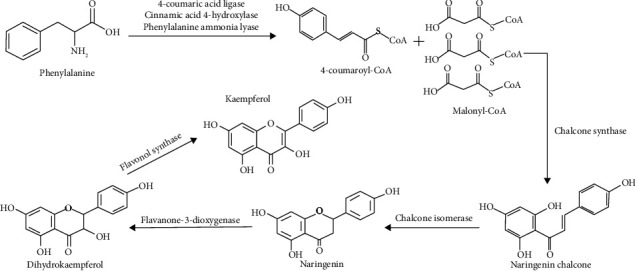
Biosynthesis of kaempferol.

Kaempferol was a compound structurally consisting of diphenylpropane, formed with condensation processes using various enzymes through the shikimate acid pathway (Figure 1). In the initial stage, phenylalanine was converted into 4-coumaroyl-CoA by 4-coumaric acid ligase, cinnamic acid 4-hydroxylase, and phenylalanine ammonia lyase. Furthermore, 3 molecules of malonyl-CoA and 1 molecule of 4-coumaroyl-CoA were used to produce naringenin through condensation reaction using the enzymes chalcone synthase and chalcone isomerase. The hydroxyl groups were then added to the C3 ring of naringenin using the flavanone-3-dioxygenase enzyme, leading to the production of dihydrokaempferol. In the final stage, the double bond at C2–C3 of dihydrokaempferol was formed using the flavonol synthase enzyme, thereby producing kaempferol [17, 18].

## 3. Pathophysiology and Epidemiology of Kidney Diseases

Several studies have reported that several factors contributed to the occurrence of kidney diseases, including fibrosis, glomerulosclerosis, and tubulointerstitial fibrosis, leading to a reduction in the organ's function (renal failure) [19]. The primary causes were immunological reaction, hypoxia, tissue ischemia, exogenous agents such as drugs, endogenous substances such as glucose, and genetic disorders [20]. In terms of pathophysiology, several aspects must be considered, including the structural and physiological characteristics of the kidney, as well as the principles of kidney tissue injury and repair. The renal blood flow rate was in the range of 400 mL/100 g of tissue/minute, which was higher compared to well-perfused vascular vessels in other organs, such as the heart, brain, and liver. Consequently, the tissues of the organ could be exposed to a large amount of circulating agents or substances that had the potential to cause damage. The glomerular filtration, which depended on high intra- and trans-glomerular pressure, could make glomerular capillaries vulnerable to hemodynamic injuries. This could lead to hypertension and hyperfiltration, which were the primary causes of chronic kidney disease (CKD). Negatively charged molecules on the glomerular filtration membrane acted as barriers for anionic macromolecules. However, disturbances in this electrostatic barrier could lead to plasma proteins moving toward the glomerular filtrate, causing injury to the glomerulus. In kidney diseases, the downstream position of tubules connected to the glomerulus and nephron microvasculature maintained glomerulotubular balance, facilitated the spread of glomerular injury to the tubulointerstitial compartment, and exposed tubular epithelial cells to abnormal ultrafiltrate. Several studies have shown that peritubular blood vessels in the glomerulus caused inflammatory reaction mediators to overflow into the peritubular circulation, leading to inflammation in the interstitial space. Any decrease in preglomerular or glomerular perfusion could also decrease blood flow, thereby causing hypoxia, tubulointerstitial injury, and kidney tissue remodeling. In normal kidney function, the glomerulus was a functional unit with its constituents, such as mesangial, endothelial, visceral, podocyte cells, and extracellular matrix. When there was damage to one part of the glomerulus, other parts were often affected through various mechanisms, including changes in matrix composition, the release of inflammatory mediators (chemokines and cytokines), basal membrane alterations, direct cell-cell connections, and growth factors. In the end, these reactions will cause a decrease in kidney function accompanied by a decrease in the GFR, resulting in excessive retention of nitrogenous waste products which in the final stage causes renal failure [21].

Kidney disease had been reported as the ninth leading cause of death globally, with almost 1.2 million people dying due to kidney failure [22]. Recent data reported that 9.1% to 13.4% of the world's population (between 700 million and 1 billion people) experienced CKD [23]. Furthermore, the high prevalence of this condition was primarily attributed to diabetes mellitus and hypertension [5]. Several drugs (aminoglycoside antibiotics, nonsteroidal anti-inflammatory drugs (NSAIDs), and chemotherapy agents), chemicals (CCl_4_, ethylene glycol, and Na_2_C_2_O_4_), and heavy metals (Pb, Hg, Cd, and As) had been reported to cause a decline in kidney function, leading to acute kidney failure, chronic interstitial nephritis, and nephrotic syndrome [24]. At present, there are over 1 million people worldwide in need of dialysis or transplantation [4]. According to previous studies, transplantation was the only therapeutic option for end-stage kidney failure patients. For patients unable to undergo the option, dialysis became an alternative management method [25].

## 4. Potential Nephroprotective Effects of Kaempferol

At present, several studies have reported that kaempferol exhibited significant pharmacological activities, including anti-inflammatory, antitumor, antimalarial, antioxidant, and hepatoprotective [13, 26–29]. However, some studies had also shown the nephroprotective potential of kaempferol in both *in vitro* and *in vivo* models [30]. This compound also had the ability to reduce kidney damage in cisplatin-treated mice by decreasing the levels of ROS, IL-12, and TNF-*α*, as well as reducing apoptosis through the inhibition of MAPK and NF-*κ*B and upregulating Nrf-2/HO-1 levels [30]. Kaempferol also showed nephroprotective effects in streptozotocin-induced diabetic nephropathy rats by enhancing insulin secretion, inhibiting NF-*κ*B p65, increasing Bcl2 protein levels, reducing total levels of caspase-3, Bax, p38 MAPK, p-JNK, and cytochrome-c in the cytoplasm, as well as downregulating TRAF6 expression [31, 32]. A previous study stated that it could act as an antifibrotic agent for kidney fibrosis by activating the BMP-7-Smad1/5 signaling pathway [33]. The compound also inhibited invasion and migration of RCC by decreasing regulation in the AKT and FAK pathways [34], inhibiting the activation of the EGFR/p38 signaling pathway, reducing cyclin B1 expression regulation, inducing apoptosis, and inhibiting RCC growth [35]. Various studies reporting the role of kaempferol in managing renal injuries are summarized in Table 1.

### 4.1. Kaempferol Ameliorates Renal Injury by Increasing Antioxidants, Reducing Inflammation, and Suppressing Apoptosis

Kidney played an essential role in human physiology, including maintaining fluid homeostasis, regulating blood pressure, producing erythrocytes and bone tissues, regulating hormone balance, and filtering nitrogen and other waste products [5]. However, the organ was also vulnerable to toxic chemical compounds, leading to AKI [30]. Sustained injury could lead to glomerulosclerosis, tubulointerstitial fibrosis, and tubular cell apoptosis, thereby causing kidney failure [48]. Kidney disease was the most vital health problem, and several drugs were implicated in this condition [25]. A typical example was the use of cisplatin (CPL), which has been proven to induce injury to the organ [30]. CPL-mediated nephrotoxicity was reported to be associated with several pathways [49], including increasing the production of ROS, nitrite, and iNOS in kidney tubules, leading to damage [49, 50]. Furthermore, increased ROS production could trigger signaling cascades, such as MAPK, p53, and NF-*κ*B, enhancing its damaging effects [51]. CPL was also reported to cause the release of IL-12, TNF-*α*, and IL-1*β*, leading to leukocyte infiltration within 72 hours of kidney damage [52]. The compound could induce the phosphorylation of I*κ*B*α* by activating NF-*κ*B and translocating it to the nucleus, where this could trigger genes related to inflammation, such as cytokines, COX-2, and iNOS [53, 54]. This inflammatory process was an essential mechanism in the development of CPL-mediated kidney injury [55]. According to a previous study, CPL significantly increased the levels of p38, pERK1/2, and JNK in kidney tissues after 72 hours. The activation of ERK1/2 was known to induce apoptosis in kidney cells through the p53 and Bax pathways [30, 49, 51, 56, 57].

**Figure 2:**
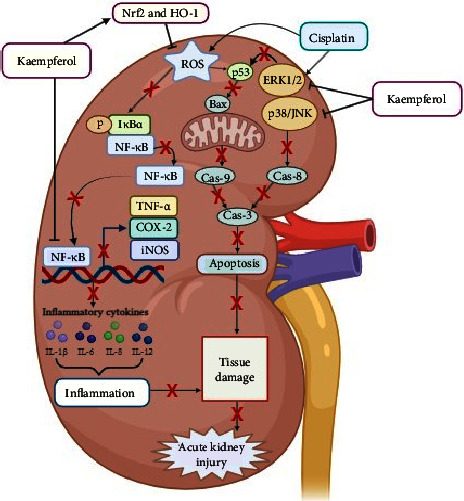
Kaempferol could protect the CPL-induced AKI by increasing antioxidants, reducing inflammation, and suppressing apoptosis. Inhibition (⟞) and red crosses (suppression).

Kaempferol was reported to reduce the formation of ROS and induce reparative effects on superoxide anion, hydroxyl radicals, and peroxynitrite [58, 59]. Furthermore, it enhanced the activity of antioxidant enzymes, such as catalase and superoxide dismutase [60]. Several studies showed that its significant administration increased Nrf-2 and HO-1 levels [30]. Nrf-2 was a protein responsible for regulating the expression of antioxidant proteins that prevented oxidative damage from injury and inflammation [61, 62], while HO-1 was a gene with a role in balancing oxidative stress [63]. Kaempferol had also been shown to reduce the release of proinflammatory cytokines (IL-12 and TNF-*α*) and inhibit NF-*κ*B, thereby reducing inflammation in CPL-induced kidney injury. The compound also had the ability to inhibit the activation of p38, ERK, and JNK as well as decrease CPL-mediated p53 levels, thereby suppressing cell death. Therefore, it could alleviate CPL-mediated kidney injury by reducing oxidative stress (ROS) and nitryl ion levels, enhancing Nrf-2 and HO-1, and reducing inflammation through the inhibition of proinflammatory cytokine release (IL-12 and TNF-*α*) and NF-*κ*B. These effects were also achieved by suppressing apoptosis through the inhibition of p38, ERK, and JNK activation, as well as reducing p53 levels (Figure 2) [30].

### 4.2. Kaempferol Suppresses Renal Damage by Activating SIRT1 Signaling

Approximately 10% of the world's population suffers from CKD with a high mortality rate [64]. This condition was characterized by the progressive loss of kidney function with damage to glomeruli, tubules, and blood vessels [65]. Several studies had reported that SIRT1 played an essential role in preventing CKD by reducing oxidative stress, inflammation, fibrosis [66–69], and apoptosis [70]. SIRT1 was a protein expressed in the kidney, which protected and maintained the normal function of the organ's cells by mediating various physiological processes [71]. The protein also functioned as an NAD^+^-dependent enzyme that promoted cell survival, antioxidant synthesis, and mitochondrial biogenesis [72]. Furthermore, it could inhibit TNF-*α* and inflammation by deacetylating NF-*κ*B, p65, and p53 [73], leading to reduced cytokine production in fibroblast cells and inhibition of several proinflammatory gene expressions [74]. SIRT1 had been reported to play a role in the process of kidney fibrosis, a key feature of progressive CKD [75]. A previous study stated that its deficiency in endothelial cells increased peritubular capillary rarefaction [76] and worsened nephrosclerosis, through decreased regulation of matrix metalloproteinase-14 (MMP-14) [77]. The protein was also known to regulate the process of fibrosis by inducing the deacetylation of Smad4 and inhibiting the expression of TGF-*β*-mediated MMP-7 in renal tubular epithelial cells [78]. Activation of SIRT1 weakened the fibrotic process in the kidney by reversing the acetylation of Smad3, causing the inhibition of the profibrotic response of TGF-*β*1 [79, 80]. Specific tubular cell overexpression mitigated the transition from acute AKI to CKD by deacetylating Smad4 [78]. SIRT1 was also reported to suppress the expression of Bax through direct deacetylation of p53 after DNA damage and oxidative stress, while deacetylating FOXO3a to stimulate antioxidant synthesis and antiapoptotic protein production [73].

Previous studies showed that the use of drugs, such as doxorubicin (DOX), suppressed SIRT1 signaling and significantly reduced its activity [81–83]. These activities increased the production of oxidative stress (ROS) and inflammatory cytokines through complex interconnected mechanisms. Mechanisms comprised the reduction of iron ions, activation of several ROS-producing enzymes (such as NQO1), NADPH reductase, NOS-3, and XDH, depletion of GSH stores, suppression of Nrf-2 (HO-1), and activation of NF-*κ*B. This created a complex vicious cycle of activation between ROS, NF-*κ*B, and Nrf-2 [84–88]. Meanwhile, increased oxidative stress and inflammation could lead to fibrosis activation by stimulating various pathways in kidney cells, including TGF-*β*1/Smad3, p53, Bax, p38 MAPK, JNK/SAPK, and caspase-1/3 [89, 90]. DOX administration was reported to cause intrinsic cell death characterized by upregulation of Bax, downregulation of Bcl-2, increased mitochondrial permeability, and caspase-3 activation in kidney cells [90, 91].

**Figure 3:**
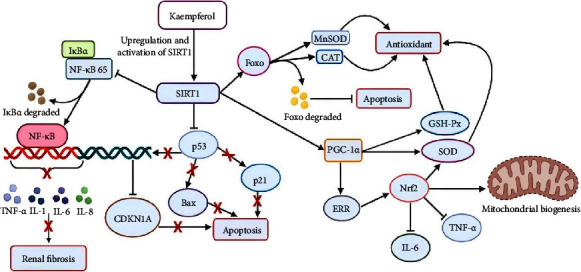
Kaempferol suppresses renal damage by the upregulation and activation of SIRT1. Inhibition (⟞) and red crosses (suppression).

In recent times, kaempferol was reported to provide broad protection against oxidative stress, inflammation, fibrosis, and apoptosis mediated by DOX in kidney tissues by activating and enhancing SIRT1 [43]. The increase in SIRT1 stimulated Nrf-2 activation while inhibiting NF-*κ*B. SIRT1 could also stimulate the expression of antioxidant and antiapoptotic genes through the deacetylation of the transcription factors FOXO and p53, thereby suppressing apoptosis. Kaempferol also reduced collagen deposition and prevented the upregulation of Bax and caspase-3. However, its nephroprotective effects reduced ROS and increased antioxidant production (such as SOD and GSH). The increase in GSH and SOD was followed by a decrease in TNF-*α* and IL-6 levels, leading to reduced kidney fibrosis (Figure 3) [43]. Based on these results, kaempferol had the potential to be developed as an antifibrotic therapy for CKD patients.

### 4.3. Kaempferol Improves Renal Function by Preventing Lipid Peroxidation (LPO)

Over the past few decades, there has been an increase in the use of nephrotoxic substances, such as therapeutic drugs, heavy metals from environmental pollution, and industrial chemicals that cause kidney damage [39]. Heavy metals were known to induce kidney injury [92], with mercury (Hg), a pollutant released into the environment through anthropogenic activities, being the most frequently encountered [93, 94]. The majority of the nephrotoxic effects of Hg accumulated in the proximal tubules [95], and excessive exposure to its compounds induced LPO [96], a chemical mechanism that disrupted the structure and function of cell membranes [97]. The cell membrane was a double-layered phospholipid with extrinsic proteins and was a direct target of LPO, which caused various harmful effects, such as osmotic fragility, increased membrane rigidity, membrane destruction, and cell damage [98]. TBARS, as one of the LPO products produced during oxidative lipid degradation, was known to play an essential role in kidney injury processes [99]. According to a previous study, Hg exposure also caused severe cellular necrosis in the kidney due to the formation of highly reactive radicals and hydroperoxide formation through phospholipid membrane peroxidation [100]. This led to increased nitrogen waste products in the urine, such as SCr, BUN, and uric acid, as well as a decrease in blood protein, as markers of dysfunction [101, 102].

The administration of kaempferol had been reported to increase the levels of antioxidant enzymes, such as SOD, CAT, and GPx, to their normal levels in tissues. This could directly prevent increased LPO by scavenging free oxygen radicals, breaking the LPO chain, reducing kidney TBARS levels, and preventing increased membrane permeability due to oxidative damage. Furthermore, the anti-LPO, antioxidant, and metal-chelating effects of kaempferol could repair pathological changes and restore kidney physiology. These effects reduced serum levels of creatinine, BUN, and uric acid and increased protein levels towards the normal level [39].

### 4.4. Kaempferol against Kidney-Damaging through Activating the Nrf2/ARE Signaling Pathway

The kidney was known to be highly susceptible to cellular injury mediated by reactive oxygen species (ROS). These compounds were produced due to intracellular catabolism reaction or exposure to exogenous toxins [103]. Furthermore, increased ROS production disrupted redox homeostasis, contributing to oxidative stress [104]. Superoxide radicals (O^2−^) were a type of ROS generated by increased regulation of NADPH oxidase [105]. Recent studies showed that NADPH oxidase-4 played an essential role in the pathogenesis of several kidney diseases and was highly expressed in the kidney [106]. However, other types of ROS, such as SOD, which catalyzed the conversion of superoxide radicals (O^2−^) into H_2_O_2_, were reported to cause injury in the organ [105]. ROS was reported to activate NF-*κ*B and increase the production of NO, TNF-*α*, IL-1*β*, IL-6, and COX-2, causing an imbalance between inflammatory factors and antioxidant defenses. This ultimately led to disturbances in the pathophysiology of various kidney injuries and diseases [107].

Cells could protect themselves from damage caused by ROS exposure by recruiting antioxidant defense systems, including SOD, CAT, and GPx. However, under certain conditions, excessively produced ROS could not be eliminated or neutralized by the antioxidant defense system [105, 108]. This situation often necessitated external antioxidants, such as kaempferol. This compound had been reported to have potent antioxidant effects in kidney damage models, both *in vitro*, *in vivo*, and clinically [48]. Kaempferol had also been shown to enhance the activation of Nrf-2, a basic-region leucine zipper (bZIP) transcription factor encoded by the nuclear factor erythroid 2 L2 (NFE2L2) gene, which regulated the expression of sequences containing antioxidant response elements (ARE) [109, 110]. Factors produced in this signaling pathway included GSH, GPx, and Trx, as well as enzymes engaged in phases one and two biotransformation of exogenous and endogenous products, NADPH regeneration, and heme metabolism. The Nrf-2/ARE signaling pathway played a crucial role in cellular detoxification and antioxidant and antiinflammatory effects [111, 112]. Increased Nrf-2/ARE interaction by kaempferol inhibited NF-*κ*B and the MAPK/ERK apoptosis cascade by reducing the release of NO, IL-12, TNF-*α*, and IL-1*β*, as well as neutralizing ROS. Moreover, this interaction reduced the regulation of TPS-three, Bax/Bcl-2, caspase-3 and 9, and PARP [103]. Increased Nrf-2 activation had also been proven to reduce tissue damage markers, such as creatinine clearance (CrCl) and BUN levels, while improving kidney function [113].

### 4.5. Selective Cytotoxicity of Kaempferol on Renal Cell Carcinoma (RCC)

RCC was a cancer disease accounting for approximately three percent of all cancers, with the highest incidence occurring in Western countries and was predicted to continue increasing [114]. In 2022, it was reported that the global death toll due to RCC was 179,368 deaths, with 115,600 and 63,768 in males and females, respectively [115]. The prevalence was 2 : 1 in males compared to females, and a higher incidence was observed in the elderly population [114–116]. RCC was the most common solid lesion in the kidney, accounting for approximately ninety percent of all kidney malignancies [117]. Furthermore, there were several histological subtypes of the condition, each marked by a unique molecular landscape, with the most common subtype being ccRCC. This subtype represented 85% of all kidney cancer cases and originated from the proximal tubule cells of the kidney nephron [118]. The prognosis for patients with metastatic ccRCC was very poor, with <10 percent of patients surviving for five years after diagnosis [119]. At present, nephrectomy and ablative therapy were often performed when kidney transplantation was not accessible, and recurrence occurred, which was the main cause of death after nephrectomy for this type of cancer [120]. Another therapeutic option for treating RCC was chemotherapy, but it had been reported that the condition exhibited high resistance to several chemotherapy agents [118, 121].

**Figure 4:**
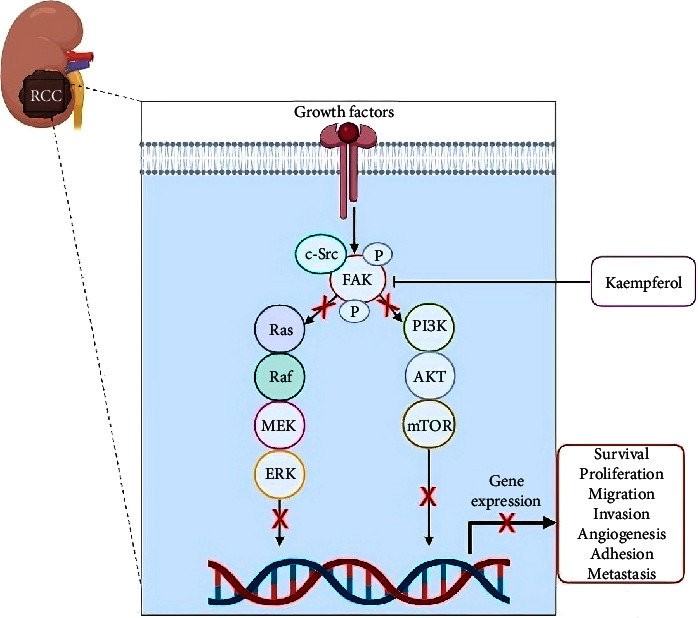
Kaempferol inhibits the growth and viability of RCC through the downregulation of FAK pathways. Inhibition (⟞) and red crosses (suppression).

The current treatment of RCC remained a significant challenge in the medical field, showing that it was crucial to discover effective natural remedies with anti-RCC effects [122]. Flavonoid compounds had gained remarkable consideration in treating various cancers, including RCC. Some studies showed that flavonoids present in medicinal plants and consumables played a crucial role in preventing the condition [123]. A common dietary flavonoid found in various fruits, vegetables, and medicinal plants was kaempferol [124, 125]. This compound had been proven to have anticancer effects by modulating apoptosis, MAPK/ERK1/2 and P13K/Akt/mTOR signaling pathways, and the VEGF signaling pathway [126, 127]. Furthermore, it was reported to be less toxic to normal cells compared to standard chemotherapy drugs [127, 128]. Kaempferol was also known to prevent RCC growth through the apoptosis pathway in RCC cell lines, such as 769-P and 786-O [35, 129]. This could induce G2-M cell cycle arrest, as well as downregulate signaling pathways, such as PI3K-PKB, and expression of EMT markers, including SNAI1, E-cadherin, N-cadherin, and MMP2 [130, 131]. The compound could stimulate the activation of caspase-9, 7, and 3, and PARP in the initiation and execution of cell apoptosis [132]. In a previous study, it inhibited cell invasion and migration while reducing the regulation of the focal adhesion kinase (FAK) pathway in RCC (Figure 4) [34]. Another report showed that it enhanced the regulation of p21, cyclin-B1, and cleavage of PARP and promoted the activation of the EGF/p38 receptor signaling pathway [35, 129]. Kaempferol was also known to maintain the survival of normal cells and prevent angiogenesis [133].

### 4.6. Kaempferol Attenuates Diabetic Nephropathy by Preventing Kidney Mitochondria Injury

Kidney was one of the most energy-demanding organs, using approximately 7% of the total oxygen available for the entire physiological functions of the human body [134]. This was attributed to the crucial role of mitochondria in its physiology [135]. Mitochondria were abundant in the kidney, specifically in renal tubule cells, compared to other organs [136, 137]. This organelle served as the central location for over ninety percent of ATP production in cells [138, 139]. An increase in ROS production (specifically O^2−^) during electron transfer to oxygen and a decrease in mitochondrial antioxidant enzymes (SOD and GSH) [140] could lead to oxidative stress, apoptosis, and mitochondrial dysfunction [141]. Mitochondrial ROS had been shown to inhibit several signaling pathways and hinder the function and activity of redox-dependent proteins, disrupting cell survival and health [142]. In the kidney, ROS was produced in the cortex and medulla, leading to changes in blood flow, proteinuria, inflammation, and fibrosis [143]. These compounds were also known to increase in diabetic patients, and the resulting increased production could cause complications in diabetic patients, including diabetic nephropathy [144]. Diabetic nephropathy was characterized by albumin excretion in urine, glomerular lesions, and a loss of the GFR in diabetic patients, both in T1DM (autoimmune destruction of beta cells and absolute insulin deficiency) and T2DM (relative insulin deficiency and resistance) [145]. The condition has been reported to cause redox changes due to persistent hyperglycemia and the accumulation of advanced glycation end products (AGEs) [146]. The resulting chronic inflammatory response caused deviating redox changes, glomerulosclerosis, proteinuria, albuminuria, and tubulointerstitial fibrosis [147]. However, increased ROS production due to diabetes damaged mitochondrial DNA, disrupting ATP synthesis and causing kidney cell dysfunction, such as glomerular endothelial cells, mesangial cells, and epithelial cells [148, 149]. In diabetic nephropathy conditions, there was an increase in GSH degradation and a decrease in inherent GSH synthesis, as well as a decrease in enzymatic activities of SOD and CAT [150]. Excess ROS production during this condition reduced the activities of AMPK, SIRT1, PGC1*α*, and mitochondrial energy metabolism [151]. Hyperglycemia effects led to increased TNF-*α*, IL-6, ROS, and MDA and higher NF-*κ*B p65 expression [152], as well as increased apoptosis shown by elevated levels of Bax protein, caspase-3, and cytochrome c in the cytoplasm and decreased regulation of Bcl-2 [153].

**Figure 5:**
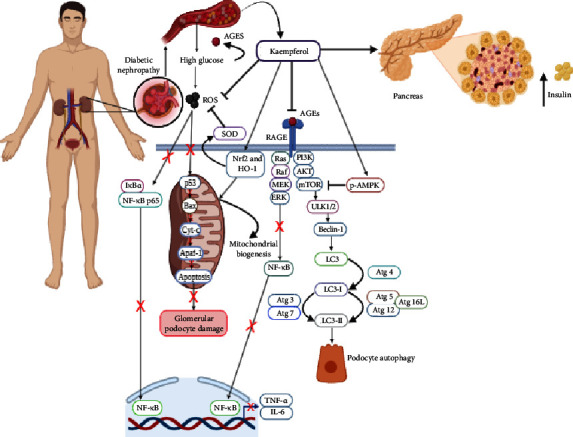
Kaempferol regulated podocyte autophagy protein expression by enhancing p-AMPK and reducing p-mTOR expression in diabetic nephropathy. Inhibition (⟞) and red crosses (suppression).

The administration of kaempferol reduced TNF-*α* and IL-6 levels in the kidney, cleft caspase-3, p38, and Bax, suppressed JNK phosphorylation, and inhibited NF-*κ*B p65 transactivation in diabetic nephropathy. Furthermore, it decreased glucose levels in diabetes, increased insulin and HOMA-*β* levels, reduced ROS and MDA levels, stimulated SOD and GSH levels, and enhanced the expression of Nrf-2 and HO-1, which were necessary for mitochondrial biogenesis [31]. The compound had also been reported to lower albuminuria levels and alleviate glycolipid metabolism dysfunction caused by diabetes. In a previous study, it mitigated mesangial matrix expansion, thickening of the glomerular basement membrane, and loss of podocyte cells. Kaempferol regulated autophagy protein expression (increasing LC3II, Beclin-1, Atg 5, and Atg 7 and decreasing p62/SQSTM1 regulation) by enhancing p-AMPK and reducing p-mTOR expression in the AMPK/mTOR signaling pathway (Figure 5) [44]. Based on these results, kaempferol had the potential to be developed as a therapeutic agent for diabetic nephropathy.

### 4.7. Clinical Prospects

Kaempferol is a flavonoid found in many plant foods, including black tea (containing 118 mg/kg of kaempferol), papaya shoots (containing 453 mg/kg of kaempferol), cauliflower (containing 270 mg/kg of kaempferol), broccoli (containing 30–72 mg/kg of kaempferol), onion leaves (containing 832 mg/kg of kaempferol), kale (containing 470 mg/kg of kaempferol), spinach (containing 550 mg/kg of kaempferol), turnip (containing 38 mg/kg of kaempferol), and beans (containing 14 mg/kg of kaempferol) [154, 155]. To show the clinical prospects of kaempferol, several clinical nutraceutical trials had been conducted on patients to test its nephroprotective effects. Gigliotti et al., and Le, TH., reported that consuming broccoli containing 30–72 mg/kg of kaempferol significantly reduced the risk of kidney failure in ARIC (atherosclerosis risk in communities) patients [156, 157]. Meanwhile, other clinical nutrition studies reported that nonsoy nut diets (containing 14 mg/kg of kaempferol) significantly reduced inflammatory biomarkers, such as CRP, IL-6, and TNF-*α*, in diabetic nephropathy patients [158]. This was consistent with Navarro et al., with a nutrition-based clinical trial on healthy adults through the administration of different cruciferous vegetables (cauliflower containing 270 mg/kg of kaempferol, broccoli containing 30–72 mg/kg of kaempferol, and turnip containing 38 mg/kg of kaempferol) observed after 14 days, and the results showed a significant decrease in IL-6 and IL-8 levels [159].

At present, clinical use of kaempferol is hindered by its low bioavailability [160]. To enhance its clinical effectiveness, innovative methods using nanotechnology were required. These methods aimed to coat the surface of its compounds to improve systemic absorption and bioavailability in the body, such as encapsulation using PLGA and PEO-PPO-PEO nanoparticle layers. However, further clinical trials were needed to evaluate kaempferol as a drug for managing various health issues, specifically as a nephroprotective agent [161].

## 5. Challenges, Limitations, and Recommendations for Future Research

There are a few challenges and limitations about kaempferol that could affect their clinical efficacy, which are their physicochemical instability, their low bioavailability and limited water solubility, and their rapid metabolism. Further explanation could be reviewed in these literature studies [13, 155]. Kaempferol has been reported to show low bioavailability and limited stability, as these natural compounds are sensitive to the degradation or transformation to inactive derivates. Consequently, this will limit their effectiveness [162]. To overcome these issues, the use of nanotechnology and nanocarrier-based approaches in the delivery of kaempferol and its derivatives may help and improve the therapeutic responses and enhance their effectiveness [163–165]. The use of nanoparticles in the delivery system can increase the bioavailability of kaempferol and its derivatives. The most common types of nanoparticles used are polymeric nanoparticles, liposomes, solid lipid nanoparticles, micelles, crystal nanoparticles, and complexes with dendrimers [166]. There have been several studies reported on the use of nanoparticles with kaempferol and its derivatives. For example, nanokaempferol developed using a layer-by-layer technique enhances the bioavailability of kaempferol in bone marrow [164], kaempferol loaded nanoparticles against hepatocellular carcinoma [167], and kaempferol nanoparticles could protect the heart against chemotherapy drugs [168].

## 6. Conclusions

In conclusion, kidney disease is a public health epidemic associated with an increased risk of mortality. Kaempferol has been reported to have various health benefits, including as a nephroprotective agent. A review of recent advances in its role and mechanisms showed that it could reduce damage to the organ. The compound also exhibited significant biological activity in kidney diseases, such as antioxidant, anti-inflammatory, antifibrotic, and antidiabetic effects. This data supported the role of kaempferol as the potential compound for further study to develop new therapeutic agents. Based on results, there are limited studies on the effects of the compound, showing that its clinical application required further investigations. Furthermore, it was crucial to determine the metabolites produced after kaempferol administration and enhance its bioavailability, which could contribute to improving its effectiveness. The development of new approaches/strategies, such as the application of nanotechnology in the delivery of kaempferol and its derivatives, that help to promote the access of nephroprotective to the kidney, is needed to boost more nephroprotection actions of kaempferol and its derivatives for the prevention and treatment of kidney diseases.

## Figures and Tables

**Table 1:** The role of kaempferol in alleviating renal diseases.

Disease types	In vitro model	In vivo model	Mechanism of action	Doses/concentrations	References
Acute kidney injury	—	Cisplatin-induced mouse nephrotoxicity	↓ Oxidative stress (ROS), inflammation (IL-12 dan TNF-*α*), and apoptosis via inhibiting MAPK and NF-*κ*B cascade and up-regulating the Nrf-2/HO-1 level	100 and 200 mg/kg	[30]
Acute kidney injury	—	Lipopolysaccharide (LPS)-induced acute kidney injury in mice	Down-regulation of HMGB1, TLR4, and P2X7 inflammatory proteins	25, 50, and 100 mg/kg	[36]
Acute kidney injury	—	Sepsis-associated acute kidney injury in the mice model	↓ Production of MCP-1, VCAM-1, and ICAM-1 and macrophage infiltration	1 mg/kg	[37]
Acute kidney injury	HK-2 cells	Sepsis-induced acute kidney injury in C57BL/6 mice	Inhibition of the NF-*κ*B/AKT signaling pathway	25, 50, and 100 mg/kg and 5, 10, and 20 *µ*M	[38]
Nephropathy	—	Mercuric chloride (HgCl_2_)-induced nephrotoxicity in wistar albino rats	↑ Production of antioxidant enzymes (SOD, CAT, and GPx) and decreased level of TBARS, creatinine, blood urea nitrogen, and uric acid	100 mg/kg	[39]
Nephropathy	NRK-52E and RPTEC cells	—	Inhibition of RhoA/Rho-kinase-mediated inflammatory signaling	5, 10, and 50 *μ*M	[40]
Nephropathy	D-ribose-induced mesangial cell injury (SV40 MES13 cells)	—	Reduced apoptosis via the mitochondria-dependent caspase-9/3 pathway, inhibition of AGE formation, attenuated oxidative ROS production, and protected mitochondrial membrane integrity	1, 2, and 5 *μ*M	[41]
Nephropathy	HEK-293T and NRK-52E cells	Streptozotocin-induced diabetic nephropathy in rats, C57BL/6 mice	↑ Insulin-releasing, upregulation of Nrf-2 and HO-1, inhibits NF-*κ*B p65, increases levels of Bcl2, and decreases in total protein levels of cleaved caspase-3, Bax, p38 MAPK, p-JNK, and cytoplasmic levels of cytochrome-c, and downregulation of TRAF6 expression	10 and 200 mg/kg, and 2.5 *μ*M	[31, 32]
Nephropathy	—	Cadmium chloride (CdCl_2_)-induced nephropathy in rats	Suppression of NF-*κ*B p65, down-regulation of Keap1, and trans-activating of Nrf2	200 mg/kg	[42]
Nephropathy	—	Doxorubicin-mediated nephropathy in rats	Upregulation and activation of SIRT1 signaling	200 mg/kg	[43]
Nephropathy	—	Type-2 diabetic nephropathy model of db/db mice	Reduced apoptosis and enhanced podocytes autophagy through AMPK/mTOR pathways	50 and 100 mg/kg	[44]
Nephropathy	NRK-52E cells	Doxorubicin-induced renal tubular injury in BALB/c mice	Inhibition of the ROS/ASK1-mediated MAPK signaling pathway	10 mg/kg and 100 *μ*M	[45]
Renal fibrosis	GLUTag and MIN6 cells	Diabetes-induced fibrosis and renal damage in C57BL/6 mice	↓ Blood glucose levels by increasing the GLP-1 or insulin release, ↓ expression levels of TGF-*β*1, CTGF, fibronectin, collagen IV, IL-1*β*, RhoA, ROCK2, and p-MYPT1	100 and 200 mg/kg and 1, 5, 10, and 50 *μ*M	[46]
Renal fibrosis	NRK-52E cells	UUO-induced renal fibrosis in Sprague-Dawley rats	Activation of the BMP-7-Smad1/5 signaling pathway	30, 60, and 90 mg/kg, and 0.5, 1, 5, 10, 20, 40, 80, 100, and 200 *μ*M	[33]
Renal cell carcinoma	786-O and 769-P cells	—	Inhibition of activation of EGFR/p38 signaling pathways, upregulation of p21 expression, and downregulation of cyclin B1 expression, activation of PARP cleavages, induction of apoptosis, and inhibition of cell growth	50, 100, and 150 *μ*M	[35, 47]
Renal cell carcinoma	786-O and HK-2 cells	—	Inhibition of invasion and migration of renal cancer cells (RCC) through the downregulation of AKT and FAK pathways	25, 50, 75, and 100 *μ*M	[34]

## Data Availability

The data used in this study are available within the article.

## Abbreviations

CPL: Cisplatin
LPO: Lipid peroxidation
ROS: Reactive oxygen species
RCC: Renal cell carcinoma
GFR: Glomerular filtration rate
BUN: Blood urea nitrogen
SLE: Systemic lupus erythematosus
UTI: Urinary tract infection
CKD: Chronic kidney disease
NSAIDs: Nonsteroidal anti-inflammatory drugs
CCl_4_: Carbon tetrachloride
Na_2_C_2_O_4_: Sodium oxalate
Pb: Lead
Hg: Mercury
Cd: Cadmium
As: Arsenic
ILs: Interleukins
TNF-*α*: Tumor necrosis factor-alpha
MAPK: Mitogen-activated protein kinase
NF-*κ*B: Nuclear factor kappa B
Nrf2: Nuclear factor-erythroid-2-related factor 2
HO-1: Heme oxygenase 1
Bcl2: B-cell lymphoma protein 2
Bax: Bcl-2-associated X protein
p38: Protein 38
p-JNK: Phospho c-jun N-terminal kinase
TRAF6: TNF receptor-associated factor 6
BMP-7-Smad1/5: Bone morphogenic protein-7-suppressor of mothers against decapentaplegic 1/5
AKT: Protein kinase B
FAK: Focal adhesion kinase
EGFR: Epidermal growth factor receptor
AKI: Acute kidney injury
I*κ*B*α*: Inhibitor of kappa B alpha
COX-2: Cyclooxygenase-2
iNOS: Inducible nitric oxide synthase
pERK1/2: Phospho extracellular signal-regulated kinase 1/2
p53: Protein 53
SIRT1: Silent mating type information regulation 2 homolog-1
NAD+: Nicotinamide adenine dinucleotide
p65: Protein 65
MMPs: Matrix metalloproteinases
TGF-*β*: Transforming growth factor beta
DNA: Deoxyribonucleic acid
FOXO: Forkhead box O
DOX: Doxorubicin
NQO1: NAD(P)H quinone dehydrogenase 1
NADPH: Nicotinamide adenine dinucleotide phosphate
NOS: Nitric oxide synthase
XDH: Xanthine dehydrogenase
GSH: Glutathione
SAPK: Stress-activated protein kinase
SOD: Superoxide dismutase
CDKN1A: Cyclin-dependent kinase inhibitor 1A
p21: Protein 21
MnSOD: Manganese superoxide dismutase
CAT: Catalase
GSH-Px: Plasma glutathione peroxidase
PGC-1*α*: Peroxisome proliferator-activated receptor-gamma coactivator-1 alpha
ERR: Estrogen-related receptors
TBARS: Thiobarbituric acid reactive substances
SCr: Serum creatinine
GPx: Glutathione peroxidase
ARE: Antioxidant response element
O^2−^: Superoxide radicals
H_2_O_2_: Hydrogen peroxide
bZIP: Basic-region leucine zipper
NFE2L2: Nuclear factor erythroid 2 L2
TPS3: Trehalose 6-phosphate synthase
PARP: Poly ADP-ribose polymerase
CrCl: Creatinine clearance
ccRCC: Clear cell renal cell carcinoma
PI3Ks: Phosphoinositide 3-kinases
mTOR: Mammalian target of rapamycin
VEGF: Vascular endothelial growth factor
PI3K-PKB: Phosphatidylinositol 3-kinase protein kinase B
EMT: Epithelial-mesenchymal transition
ATP: Adenosine triphosphate
Src: Proto-oncogene tyrosine-protein kinase
T1DM: Type 1 diabetes
AGEs: Advanced glycation end products
AMPK: AMP-activated protein kinase
PGC1*α*: Peroxisome proliferator-activated receptor gamma coactivator 1- alpha
HOMA-*β*: Homeostasis model assessment beta
LC: Microtubule-associated protein light chain
Atg: Autophagy related
p62: Protein 62
SQSTM1: Sequestosome 1
Cyt c: Cytochrome c
Apaf-1: Apoptotic protease activating factor 1
ULK 1/2: Unc-51-like kinase 1/2
ARIC: Atherosclerosis risk in communities
CRP: C-reactive protein
PLGA: Polylactic acid-co-glycolic acid
PEO-PPO-PEO: Polyethylene oxide-polypropylene oxide-polyethylene oxide
HMGB1: High mobility group box 1
TLR4: Toll-like receptor 4
P2X7: Purinoceptor 7
MCP-1: Monocyte chemoattractant protein-1
VCAM-1: Vascular cell adhesion molecule 1
ICAM-1: Intercellular adhesion molecule 1
ASK1: Apoptosis signal-regulating kinase 1
GLP-1: Glucagon-like peptide-1
CTGF: Connective tissue growth factor
ROCK2: Rho-associated coiled-coil containing protein kinase 2
p-MYPT1: Phospho myosin phosphatase target subunit 1.

## References

[1] Abdel-Hady H., El-Sayed M. M., Abdel-Hady A. A. (2018). Nephroprotective activity of methanolic extract of *Lantana camara* and squash (*Cucurbita pepo*) on cisplatin-induced nephrotoxicity in rats and identification of certain chemical constituents of *Lantana camara* by HPLC-ESIMS. *Pharmacognosy Journal*.

[2] Al-Snafi A. E., Talab T. A. (2019). A review of medicinal plants with nephroprotective effects. *GSC Biological and Pharmaceutical Sciences*.

[3] Sujana D., Saptarini N. M., Sumiwi S. A., Levita J. (2021). Nephroprotective activity of medicinal plants: a review on in-silico, in-vitro, and in-vivo based studies. *Journal of Applied Pharmaceutical Science*.

[4] Wannes W. A., Tounsi M. S. (2023). Tunisian nephroprotective plants: a review. *Journal of Exploratory Research in Pharmacology*.

[5] Tienda-Vázquez M. A., Morreeuw Z. P., Sosa-Hernández J. E. (2022). Nephroprotective plants: a review on the use in pre-renal and post-renal diseases. *Plants*.

[6] Chawla L. S., Bellomo R., Bihorac A. (2017). Acute kidney disease and renal recovery: consensus report of the acute disease quality initiative (ADQI) 16 workgroup. *Nature Reviews Nephrology*.

[7] Kdigo (2012). Kidney disease: improving global outcomes (KDIGO) acute kidney injury workgroup. KDIGO clinical practice guideline for acute kidney injury. *Kidney International Supplements*.

[8] Manzoor H., Bhatt H. (2020). *Prerenal Kidney Failure*.

[9] Matuszkiewicz-Rowińska J., Małyszko J. (2020). Acute kidney injury, its definition, and treatment in adults: Guidelines and reality. *Polish Archives of Internal Medicine*.

[10] Makris K., Spanou L. (2016). Acute kidney injury: definition, pathophysiology and clinical phenotypes. *Clinical Biochemist Reviews*.

[11] Lawson S. K., Satyal P., Setzer W. N. (2021). The volatile phytochemistry of seven native american aromatic medicinal plants. *Plants*.

[12] Sabiu S., O’Neill F. H., Ashafa A. O. T. (2016). The purview of phytotherapy in the management of kidney disorders: a systematic review on Nigeria and South Africa. *African Journal of Traditional, Complementary and Alternative Medicines*.

[13] Alkandahri M. Y., Pamungkas B. T., Oktoba Z. (2023). Hepatoprotective effect of kaempferol: a review of the dietary sources, bioavailability, mechanisms of action, and safety. *Advances in Pharmacological and Pharmaceutical Sciences*.

[14] Imran M., Salehi B., Sharifi-Rad J. (2019). Kaempferol: a key emphasis to its anticancer potential. *Molecules*.

[15] Sharifi-Rad M., Fokou P. V. T., Sharopov F. (2018). Antiulcer agents: From plant extracts to phytochemicals in healing promotion. *Molecules*.

[16] Alkandahri M. Y., Yuniarsih N., Berbudi A., Subarnas A. (2022). Antimalaria activities of several active compounds from medicinal plants. *Pharmacognosy Journal*.

[17] Calderón-Montaño J. M., Burgos-Morón E., Pérez-Guerrero C., López-Lázaro M. (2011). A review on the dietary flavonoid kaempferol. *Mini-Reviews in Medicinal Chemistry*.

[18] Duan L., Ding W., Liu X. (2017). Biosynthesis and engineering of kaempferol in *Saccharomyces cerevisiae*. *Microbial Cell Factories*.

[19] Fine L. G., Norman J. T. (2008). Chronic hypoxia as a mechanism of progression of chronic kidney diseases: from hypothesis to novel therapeutics. *Kidney International*.

[20] Schlondorff D. O. (2008). Overview of factors contributing to the pathophysiology of progressive renal disease. *Kidney International*.

[21] Matovinović M. S. (2009). Pathophysiology and classification of kidney diseases. *Electronic Journal of the International Federation of Clinical Chemistry and Laboratory Medicine*.

[22] Luyckx V. A., Tonelli M., Stanifer J. W. (2018). The global burden of kidney disease and the sustainable development goals. *Bulletin of the World Health Organization*.

[23] Sundström J., Bodegard J., Bollmann A. (2022). Prevalence, outcomes, and cost of chronic kidney disease in a contemporary population of 2·4 million patients from 11 countries: The CaReMe CKD study. *Lancet Regional Health-Europe*.

[24] Iqbal S. M., Hussain L., Hussain M. (2022). Nephroprotective potential of a standardized extract of *Bambusa arundinacea*: In vitro and in vivo studies. *ACS Omega*.

[25] Sundararajan R., Bharampuram A., Koduru R. (2014). A review on phytoconstituents for nephroprotective activity. *Pharmacophore*.

[26] Wang J., Fang X., Ge L. (2018). Antitumor, antioxidant and anti-inflammatory activities of kaempferol and its corresponding glycosides and the enzymatic preparation of kaempferol. *PLoS One*.

[27] Sharma N., Biswas S., Al-Dayan N., Alhegaili A. S., Sarwat M. (2021). Antioxidant role of kaempferol in prevention of hepatocellular carcinoma. *Antioxidants*.

[28] Alkandahri M. Y., Patala R., Berbudi A., Subarnas A. (2021). Antimalarial activity of curcumin and kaempferol using structure based drug design method. *Journal of Advanced Pharmacy Education & Research*.

[29] Alkandahri M. Y., Arfania M., Abriyani E. (2022). Evaluation of antioxidant and antipyretic effects of ethanolic extract of cep-cepan leaves (*Castanopsis costata* (Blume) A.DC). *Journal of Advanced Pharmacy Education & Research*.

[30] Wang Z., Sun W., Sun X., Wang Y., Zhou M. (2020). Kaempferol ameliorates cisplatin induced nephrotoxicity by modulating oxidative stress, inflammation and apoptosis via ERK and NF-*κ*B pathways. *AMB Express*.

[31] Alshehri A. S. (2021). Kaempferol attenuates diabetic nephropathy in streptozotocin-induced diabetic rats by a hypoglycaemic effect and concomitant activation of the Nrf-2/Ho-1/antioxidants axis. *Archives of Physiology and Biochemistry*.

[32] Luo W., Chen X., Ye L. (2021). Kaempferol attenuates streptozotocin-induced diabetic nephropathy by downregulating TRAF6 expression: The role of TRAF6 in diabetic nephropathy. *Journal of Ethnopharmacology*.

[33] Ji X., Cao J., Zhang L., Zhang Z., Shuai W., Yin W. (2020). Kaempferol protects renal fibrosis through activating the BMP-7-Smad1/5 signaling pathway. *Biological and Pharmaceutical Bulletin*.

[34] Hung T. W., Chen P. N., Wu H. C. (2017). Kaempferol inhibits the invasion and migration of renal cancer cells through the downregulation of AKT and FAK pathways. *International Journal of Medical Sciences*.

[35] Song W., Dang Q., Xu D. (2014). Kaempferol induces cell cycle arrest and apoptosis in renal cell carcinoma through EGFR/p38 signaling. *Oncology Reports*.

[36] Xiao C., Ying-lin Y., Wei-han L., Man L., Yue-hua W., Guan-hua D. (2020). Effect and possible mechanism of kaempferol on acute kidney injury in LPS-stimulated mice. *Chinese Pharmaceutical Journal*.

[37] Xu Z., Wang X., Kuang W., Wang S., Zhao Y. (2023). Kaempferol improves acute kidney injury via inhibition of macrophage infiltration in septic mice. *Bioscience Reports*.

[38] Chen D., Ma S., Ye W. (2023). Kaempferol reverses acute kidney injury in septic model by inhibiting NF-*κ*B/AKT signaling pathway. *Journal of Food Biochemistry*.

[39] Vijayaprakasha S., Langeswaran K., Kumar S. G., Revathya R., Balasubramaniana M. P. (2013). Nephro-protective significance of kaempferol on mercuric chloride induced toxicity in wistar albino rats. *Biomedicine and Aging Pathology*.

[40] Sharma D., Gondaliya P., Tiwari V., Kalia K. (2019). Kaempferol attenuates diabetic nephropathy by inhibiting RhoA/Rho-kinase mediated inflammatory signalling. *Biomedicine & Pharmacotherapy*.

[41] Zhang N., Zhao S., Hong J., Li W., Wang X. (2019). Protective effects of kaempferol on D-ribose-induced mesangial cell injury. *Oxidative Medicine and Cellular Longevity*.

[42] Alshehri A. S., El-Kott A. F., El-Kenawy A. E. (2022). The ameliorative effect of kaempferol against CdCl_2_-mediated renal damage entails activation of Nrf2 and inhibition of NF-kB. *Environmental Science and Pollution Research*.

[43] Alagal R. I., AlFaris N. A., Alshammari G. M., Altamimi J. Z., AlMousa L. A., Yahya M. A. (2022). Kaempferol attenuates doxorubicin-mediated nephropathy in rats by activating SIRT1 signaling. *Journal of Functional Foods*.

[44] Sheng H., Zhang D., Zhang J. (2022). Kaempferol attenuated diabetic nephropathy by reducing apoptosis and promoting autophagy through AMPK/mTOR pathways. *Frontiers of Medicine*.

[45] Wu Q., Chen J., Zheng X. (2023). Kaempferol attenuates doxorubicin-induced renal tubular injury by inhibiting ROS/ASK1-mediated activation of the MAPK signaling pathway. *Biomedicine & Pharmacotherapy*.

[46] Sharma D., Tekade R. K., Kalia K. (2020). Kaempferol in ameliorating diabetes-induced fibrosis and renal damage: An in vitro and in vivo study in diabetic nephropathy mice model. *Phytomedicine*.

[47] Rajendran P., Rengarajan T., Nandakumar N., Palaniswami R., Nishigaki Y., Nishigaki I. (2014). Kaempferol, a potential cytostatic and cure for inflammatory disorders. *European Journal of Medicinal Chemistry*.

[48] Alsawaf S., Alnuaimi F., Afzal S. (2022). Plant flavonoids on oxidative stress-mediated kidney inflammation. *Biology*.

[49] Kang K. P., Park S. K., Kim D. H. (2011). Luteolin ameliorates cisplatin-induced acute kidney injury in mice by regulation of p53-dependent renal tubular apoptosis. *Nephrology Dialysis Transplantation*.

[50] Zirak M. R., Rahimian R., Ghazi-Khansari M. (2014). Tropisetron attenuates cisplatin-induced nephrotoxicity in mice. *European Journal of Pharmacology*.

[51] Sung M. J., Kim D. H., Jung Y. J. (2008). Genistein protects the kidney from cisplatin-induced injury. *Kidney International*.

[52] Guerrero-Beltrán C. E., Mukhopadhyay P., Horváth B. (2012). Sulforaphane, a natural constituent of broccoli, prevents cell death and inflammation in nephropathy. *Journal of Nutritional Biochemistry*.

[53] Park M. J., Lee E. K., Heo H. S. (2009). The anti-inflammatory effect of kaempferol in aged kidney tissues: The involvement of nuclear factor-kappaB via nuclear factor-inducing kinase/IkappaB kinase and mitogen-activated protein kinase pathways. *Journal of Medicinal Food*.

[54] Yu X., Meng X., Xu M. (2018). Celastrol ameliorates cisplatin nephrotoxicity by inhibiting NF-*κ*B and improving mitochondrial function. *EBioMedicine*.

[55] Pulli B., Ali M., Forghani R. (2013). Measuring myeloperoxidase activity in biological samples. *PLoS One*.

[56] Liu Y., Song H., Song H., Feng X., Zhou C., Huo Z. (2019). Targeting autophagy potentiates the anti-tumor effect of PARP inhibitor in pediatric chronic myeloid leukemia. *AMB Express*.

[57] Wei Q., Dong G., Yang T., Megyesi J., Price P. M., Dong Z. (2007). Activation and involvement of p53 in cisplatin-induced nephrotoxicity. *American Journal of Physiology-Renal Physiology*.

[58] Wang L., Tu Y. C., Lian T. W., Hung J. T., Yen J. H., Wu M. J. (2006). Distinctive antioxidant and antiinflammatory effects of flavonols. *Journal of Agricultural and Food Chemistry*.

[59] Heijnen C. G., Haenen G. R., van Acker F. A., van der Vijgh W. J., Bast A. (2001). Flavonoids as peroxynitrite scavengers: The role of the hydroxyl groups. *Toxicology in Vitro*.

[60] Klaunig J. E., Kamendulis L. M. (2004). The role of oxidative stress in carcinogenesis. *Annual Review of Pharmacology and Toxicology*.

[61] Park H. M., Cho J. M., Lee H. R., Shim G. S., Kwak M. K. (2008). Renal protection by 3H-1,2-dithiole-3-thione against cisplatin through the Nrf2-antioxidant pathway. *Biochemical Pharmacology*.

[62] Aleksunes L. M., Goedken M. J., Rockwell C. E., Thomale J., Manautou J. E., Klaassen C. D. (2010). Transcriptional regulation of renal cytoprotective genes by Nrf2 and its potential use as a therapeutic target to mitigate cisplatin-induced nephrotoxicity. *Journal of Pharmacology and Experimental Therapeutics*.

[63] Sahin K., Tuzcu M., Gencoglu H. (2010). Epigallocatechin-3-gallate activates Nrf2/HO-1 signaling pathway in cisplatin-induced nephrotoxicity in rats. *Life Sciences*.

[64] Yan J., Wang J., He J. C., Zhong Y. (2022). Sirtuin 1 in chronic kidney disease and therapeutic potential of targeting Sirtuin 1. *Frontiers in Endocrinology*.

[65] Krisanapan P., Pattharanitima P., Thongprayoon C., Cheungpasitporn W. (2022). Recent advances in understanding of cardiovascular diseases in patients with chronic kidney disease. *Journal of Clinical Medicine*.

[66] Bordone L., Guarente L. (2005). Calorie restriction, SIRT1 and metabolism: understanding longevity. *Nature Reviews Molecular Cell Biology*.

[67] Buhrmann C., Busch F., Shayan P., Shakibaei M. (2014). Sirtuin-1 (SIRT1) is required for promoting chondrogenic differentiation of mesenchymal stem cells. *Journal of Biological Chemistry*.

[68] Hsu Y. J., Hsu S. C., Hsu C. P. (2017). Sirtuin 1 protects the aging heart from contractile dysfunction mediated through the inhibition of endoplasmic reticulum stress-mediated apoptosis in cardiac-specific Sirtuin 1 knockout mouse model. *International Journal of Cardiology*.

[69] Humphreys B. D. (2018). Mechanisms of renal fibrosis. *Annual Review of Physiology*.

[70] Liu X., Chen A., Liang Q. (2021). Spermidine inhibits vascular calcification in chronic kidney disease through modulation of SIRT1 signaling pathway. *Aging Cell*.

[71] Dong Y. J., Liu N., Xiao Z. (2014). Renal protective effect of sirtuin 1. *Journal of Diabetes Research*.

[72] Kong L., Wu H., Zhou W. (2015). Sirtuin 1: A target for kidney diseases. *Molecular Medicine*.

[73] Chong Z. Z., Shang Y. C., Wang S., Maiese K. (2012). SIRT1: New avenues of discovery for disorders of oxidative stress. *Expert Opinion on Therapeutic Targets*.

[74] Zhu X., Liu Q., Wang M. (2011). Activation of Sirt1 by resveratrol inhibits TNF-*α* induced inflammation in fibroblasts. *PLoS One*.

[75] Liu Y. (2011). Cellular and molecular mechanisms of renal fibrosis. *Nature Reviews Nephrology*.

[76] Kida Y., Zullo J. A., Goligorsky M. S. (2016). Endothelial sirtuin 1 inactivation enhances capillary rarefaction and fibrosis following kidney injury through Notch activation. *Biochemical and Biophysical Research Communications*.

[77] Vasko R., Xavier S., Chen J. (2014). Endothelial sirtuin 1 deficiency perpetrates nephrosclerosis through downregulation of matrix metalloproteinase-14: Relevance to fibrosis of vascular senescence. *Journal of American Society of Nephrology*.

[78] Simic P., Williams E. O., Bell E. L., Gong J. J., Bonkowski M., Guarente L. (2013). SIRT1 suppresses the epithelial-to-mesenchymal transition in cancer metastasis and organ fibrosis. *Cell Reports*.

[79] Liang J., Tian S., Han J., Xiong P. (2014). Resveratrol as a therapeutic agent for renal fibrosis induced by unilateral ureteral obstruction. *Renal Failure*.

[80] Huang X. Z., Wen D., Zhang M. (2014). Sirt1 activation ameliorates renal fibrosis by inhibiting the TGF-*β*/Smad3 pathway. *Journal of Cellular Biochemistry*.

[81] Ruan Y., Dong C., Patel J. (2015). SIRT1 suppresses doxorubicin-induced cardiotoxicity by regulating the oxidative stress and p38MAPK pathways. *Cellular Physiology and Biochemistry*.

[82] Song S., Chu L., Liang H. (2019). Protective effects of dioscin against doxorubicin-induced hepatotoxicity via regulation of Sirt1/FOXO1/NF-*κ*b signal. *Frontiers in Pharmacology*.

[83] Shati A. A., El-Kott A. F. (2021). Acylated ghrelin protects against doxorubicin-induced nephropathy by activating silent information regulator 1. *Basic and Clinical Pharmacology and Toxicology*.

[84] Thorn C. F., Oshiro C., Marsh S. (2011). Doxorubicin pathways: Pharmacodynamics and adverse effects. *Pharmacogenetics and Genomics*.

[85] Elsherbiny N. M., El-Sherbiny M. (2014). Thymoquinone attenuates doxorubicin-induced nephrotoxicity in rats: Role of Nrf2 and NOX4. *Chemico-Biological Interactions*.

[86] Barakat B. M., Ahmed H. I., Bahr H. I., Elbahaie A. M. (2018). Protective effect of boswellic acids against doxorubicin-induced hepatotoxicity: Impact on Nrf2/HO-1 defense pathway. *Oxidative Medicine and Cellular Longevity*.

[87] Afsar T., Razak S., Almajwal A., Al-Disi D. (2020). Doxorubicin-induced alterations in kidney functioning, oxidative stress, DNA damage, and renal tissue morphology; Improvement by *Acacia hydaspica* tannin-rich ethyl acetate fraction. *Saudi Journal of Biological Sciences*.

[88] Asaad G. F., Hassan A., Mostafa R. E. (2021). Anti-oxidant impact of lisinopril and enalapril against acute kidney injury induced by doxorubicin in male wistar rats: Involvement of kidney injury molecule-1. *Heliyon*.

[89] Ibrahim K. M., Mantawy E. M., Elanany M. M. (2020). Protection from doxorubicin-induced nephrotoxicity by clindamycin: novel antioxidant, anti-inflammatory and anti-apoptotic roles. *Naunyn-Schmiedeberg’s Archives of Pharmacology*.

[90] Lahoti T. S., Patel D., Thekkemadom V., Beckett R., Ray S. D. (2012). Doxorubicin-induced in vivo nephrotoxicity involves oxidative stress-mediated multiple pro- and anti-apoptotic signaling pathways. *Current Neurovascular Research*.

[91] Owumi S. E., Olusola J. K., Arunsi U. O., Oyelere A. K. (2021). Chlorogenic acid abates oxido-inflammatory and apoptotic responses in the liver and kidney of tamoxifen-treated rats. *Toxicology Research (Camb)*.

[92] Vukelić D., Djordjevic A. B., Anđelković M. (2023). Subacute exposure to low Pb doses promotes oxidative stress in the kidneys and copper disturbances in the liver of male rats. *Toxics*.

[93] Aleo M. F., Morandini F., Bettoni F. (2002). In vitro study of the nephrotoxic mechanism of mercuric chloride. *Medicina del Lavoro*.

[94] Peixoto N. C., Roza T., Flores E. M., Pereira M. E. (2003). Effects of zinc and cadmium on HgCl_2_-delta-ALA-D inhibition and Hg levels in tissues of suckling rats. *Toxicology Letters*.

[95] Al-Madani W. A., Siddiqi N. J., Alhomida A. S. (2009). Renal toxicity of mercuric chloride at different time intervals in rats. *Biochemistry Insights*.

[96] Ahmad S., Mahmood R. (2019). Mercury chloride toxicity in human erythrocytes: enhanced generation of ROS and RNS, hemoglobin oxidation, impaired antioxidant power, and inhibition of plasma membrane redox system. *Environmental Science and Pollution Research*.

[97] Hosohata K., Harnsirikarn T., Chokesuwattanaskul S. (2022). Ferroptosis: A potential therapeutic target in acute kidney injury. *International Journal of Molecular Sciences*.

[98] Prabu S. M., Shagirtha K., Renugadevi J. (2010). Quercetin in combination with vitamins (C and E) improves oxidative stress and renal injury in cadmium intoxicated rats. *European Review for Medical and Pharmacological Sciences*.

[99] Feng Q., Yu X., Qiao Y. (2022). Ferroptosis and acute kidney injury (AKI): Molecular mechanisms and therapeutic potentials. *Frontiers in Pharmacology*.

[100] Siddiqi N. J., Alhomida A. S. (2005). Effect of mercuric chloride on various hydroxyproline fractions in rat serum. *Molecular and Cellular Biochemistry*.

[101] Pócsi I., Dockrell M. E., Price R. G. (2022). Nephrotoxic biomarkers with specific indications for metallic pollutants: Implications for environmental health. *Biomarker Insights*.

[102] Bridges C. C., Zalups R. K. (2017). The aging kidney and the nephrotoxic effects of mercury. *Journal of Toxicology and Environmental Health-Part B: Critical Reviews*.

[103] Molaei E., Molaei A., Abedi F., Hayes A. W., Karimi G. (2021). Nephroprotective activity of natural products against chemical toxicants: The role of Nrf2/ARE signaling pathway. *Food Science and Nutrition*.

[104] Pizzino G., Irrera N., Cucinotta M. (2017). Oxidative stress: Harms and benefits for human health. *Oxidative Medicine and Cellular Longevity*.

[105] He L., He T., Farrar S., Ji L., Liu T., Ma X. (2017). Antioxidants maintain cellular redox homeostasis by elimination of reactive oxygen species. *Cellular Physiology and Biochemistry*.

[106] Meng X. M., Ren G. L., Gao L. (2018). NADPH oxidase 4 promotes cisplatin-induced acute kidney injury via ROS-mediated programmed cell death and inflammation. *Laboratory Investigation*.

[107] Liu T., Zhang L., Joo D., Sun S. C. (2017). NF-*κ*B signaling in inflammation. *Signal Transduction and Targeted Therapy*.

[108] Taherkhani S., Suzuki K., Ruhee R. T. (2021). A brief overview of oxidative stress in adipose tissue with a therapeutic approach to taking antioxidant supplements. *Antioxidants*.

[109] Liu M., Grigoryev D. N., Crow M. T. (2009). Transcription factor Nrf2 is protective during ischemic and nephrotoxic acute kidney injury in mice. *Kidney International*.

[110] Chen Q., Peng H., Dong L. (2016). Activation of the NRF2-ARE signalling pathway by the Lentinula edodes polysaccharose LNT alleviates ROS-mediated cisplatin nephrotoxicity. *International Immunopharmacology*.

[111] Iranshahy M., Iranshahi M., Abtahi S. R., Karimi G. (2018). The role of nuclear factor erythroid 2-related factor 2 in hepatoprotective activity of natural products: A review. *Food and Chemical Toxicology*.

[112] Tavakkoli A., Iranshahi M., Hasheminezhad S. H., Hayes A. W., Karimi G. (2019). The neuroprotective activities of natural products through the Nrf2 upregulation. *Phytotherapy Research*.

[113] Qin X., Meghana K., Sowjanya N. L. (2019). Embelin attenuates cisplatin-induced nephrotoxicity: Involving inhibition of oxidative stress and inflammation in addition with activation of Nrf-2/Ho-1 pathway. *BioFactors*.

[114] Capitanio U., Bensalah K., Bex A. (2019). Epidemiology of renal cell carcinoma. *European Urology*.

[115] Bukavina L., Bensalah K., Bray F. (2022). Epidemiology of renal cell carcinoma: 2022 update. *European Urology*.

[116] Tahbaz R., Schmid M., Merseburger A. S. (2018). Prevention of kidney cancer incidence and recurrence: Lifestyle, medication and nutrition. *Current Opinion in Urology*.

[117] Zhan X., Chen T., Liu Y. (2023). Trends in cause of death among patients with renal cell carcinoma in the United States: a SEER-based study. *BMC Public Health*.

[118] Makhov P., Joshi S., Ghatalia P., Kutikov A., Uzzo R. G., Kolenko V. M. (2018). Resistance to systemic therapies in clear cell renal cell carcinoma: Mechanisms and management strategies. *Molecular Cancer Therapeutics*.

[119] Turajlic S., Swanton C., Boshoff C. (2018). Kidney cancer: The next decade. *Journal of Experimental Medicine*.

[120] Kalapara A. A., Frydenberg M. (2020). The role of open radical nephrectomy in contemporary management of renal cell carcinoma. *Translational Andrology and Urology*.

[121] Brodziak A., Sobczuk P., Bartnik E. (2019). Drug resistance in papillary RCC: from putative mechanisms to clinical practicalities. *Nature Reviews Urology*.

[122] Ranjan A., Ramachandran S., Gupta N. (2019). Role of phytochemicals in cancer prevention. *International Journal of Molecular Sciences*.

[123] Bajalia E. M., Azzouz F. B., Chism D. A. (2022). Phytochemicals for the prevention and treatment of renal cell carcinoma: preclinical and clinical evidence and molecular mechanisms. *Cancers*.

[124] Alkandahri M. Y., Berbudi A., Subarnas A. (2022). Evaluation of experimental cerebral malaria of curcumin and kaempferol in *Plasmodium berghei* ANKA-infected mice. *Pharmacognosy Journal*.

[125] Alkandahri M. Y., Berbudi A., Subarnas A. (2018). Active compounds and antimalaria properties of some medicinal plants in Indonesia–a review. *Systematic Reviews in Pharmacy*.

[126] Kashafi E., Moradzadeh M., Mohamadkhani A., Erfanian S. (2017). Kaempferol increases apoptosis in human cervical cancer HeLa cells via PI3K/AKT and telomerase pathways. *Biomedicine & Pharmacotherapy*.

[127] Qattan M. Y., Khan M. I., Alharbi S. H. (2022). Therapeutic importance of kaempferol in the treatment of cancer through the modulation of cell signalling pathways. *Molecules*.

[128] Wu H., Du J., Li C., Li H., Guo H., Li Z. (2022). Kaempferol can reverse the 5-FU resistance of colorectal cancer cells by inhibiting PKM2-mediated glycolysis. *International Journal of Molecular Sciences*.

[129] Guohuan A., Gallegos J., Morris M. E. (2011). The bioflavonoid kaempferol is an Abcg2 substrate and inhibits Abcg2-mediated quercetin efflux. *Drug Metabolism and Disposition*.

[130] Imran M., Rauf A., Shah Z. A. (2019). Chemo-preventive and therapeutic effect of the dietary flavonoid kaempferol: A comprehensive review. *Phytotherapy Research*.

[131] Marfe G., Tafani M., Indelicato M. (2009). Kaempferol induces apoptosis in two different cell lines via Akt inactivation, Bax and SIRT3 activation, and mitochondrial dysfunction. *Journal of Cellular Biochemistry*.

[132] Kim K. Y., Jang W. Y., Lee J. Y. (2016). Kaempferol activates G₂-checkpoint of the cell cycle resulting in G₂-arrest and mitochondria-dependent apoptosis in human acute leukemia jurkat T cells. *Journal of Microbiology and Biotechnology*.

[133] Kim B., Jung J. W., Jung J. (2017). PGC1*α* induced by reactive oxygen species contributes to chemoresistance of ovarian cancer cells. *Oncotarget*.

[134] Vart P., Grams M. E. (2016). Measuring and assessing kidney function. *Seminars in Nephrology*.

[135] Hartogh D. J. D., Tsiani E. (2019). Health benefits of resveratrol in kidney disease: Evidence from in vitro and in vivo studies. *Nutrients*.

[136] Forbes J. M. (2016). Mitochondria-power players in kidney function?. *Trends in Endocrinology and Metabolism*.

[137] Bhargava P., Schnellmann R. G. (2017). Mitochondrial energetics in the kidney. *Nature Reviews Nephrology*.

[138] Barchiesi A., Bazzani V., Tolotto V. (2020). Mitochondrial oxidative stress induces rapid intermembrane space/matrix translocation of apurinic/apyrimidinic endonuclease 1 protein through TIM23 complex. *Journal of Molecular Biology*.

[139] Hui Y., Lu M., Han Y. (2017). Resveratrol improves mitochondrial function in the remnant kidney from 5/6 nephrectomized rats. *Acta Histochemica*.

[140] Angelova P. R., Abramov A. Y. (2018). Role of mitochondrial ROS in the brain: from physiology to neurodegeneration. *FEBS Letters*.

[141] Daenen K., Andries A., Mekahli D., Van Schepdael A., Jouret F., Bammens B. (2019). Oxidative stress in chronic kidney disease. *Pediatric Nephrology*.

[142] Galvan D. L., Green N. H., Danesh F. R. (2017). The hallmarks of mitochondrial dysfunction in chronic kidney disease. *Kidney International*.

[143] Nistala R., Whaley-Connell A., Sowers J. R. (2008). Redox control of renal function and hypertension. *Antioxidants and Redox Signaling*.

[144] Hojs N. V., Bevc S., Ekart R., Hojs R. (2020). Oxidative stress markers in chronic kidney disease with emphasis on diabetic nephropathy. *Antioxidants*.

[145] Ruiz-Ortega M., Rodrigues-Diez R. R., Lavoz C., Rayego-Mateos S. (2020). Special issue “diabetic nephropathy: Diagnosis, prevention and treatment. *Journal of Clinical Medicine*.

[146] Cepas V., Collino M., Mayo J. C., Sainz R. M. (2020). Redox signaling and advanced glycation endproducts (AGEs) in diet-related diseases. *Antioxidants*.

[147] Tiwari B. K., Pandey K. B., Abidi A. B., Rizvi S. I. (2013). Markers of oxidative stress during diabetes mellitus. *Journal of Biomarkers*.

[148] Forbes J. M., Coughlan M. T., Cooper M. E. (2008). Oxidative stress as a major culprit in kidney disease in diabetes. *Diabetes*.

[149] Fernandes S. M., Cordeiro P. M., Watanabe M., Fonseca C. D., Vattimo M. F. (2016). The role of oxidative stress in streptozotocin-induced diabetic nephropathy in rats. *Archives of Endocrinology and Metabolism*.

[150] Abdou H. M., Elkader H. A. E. A. (2022). The potential therapeutic effects of Trifolium alexandrinum extract, hesperetin and quercetin against diabetic nephropathy via attenuation of oxidative stress, inflammation, GSK-3*β* and apoptosis in male rats. *Chemico-Biological Interactions*.

[151] Rius-Pérez S., Torres-Cuevas I., Millán I., Ortega A. L., Pérez S. (2020). PGC-1*α*, inflammation, and oxidative stress: an integrative view in metabolism. *Oxidative Medicine and Cellular Longevity*.

[152] Malik S., Suchal K., Khan S. I. (2017). Apigenin ameliorates streptozotocin-induced diabetic nephropathy in rats via MAPK-NF-*κ*B-TNF-*α* and TGF-*β*1-MAPK-fibronectin pathways. *American Journal of Physiology - Renal Physiology*.

[153] Altamimi J. Z., AlFaris N. A., Alshammari G. M. (2021). Ellagic acid protects against diabetic nephropathy in rats by regulating the transcription and activity of Nrf2. *Journal of Functional Foods*.

[154] Alam W., Khan H., Shah M. A., Cauli O., Saso L. (2020). Kaempferol as a dietary anti-infammatory agent: current therapeutic standing. *Molecules*.

[155] Dabeek W. M., Marra M. V. (2019). Dietary quercetin and kaempferol: bioavailability and potential cardiovascular-related bioactivity in humans. *Nutrients*.

[156] Gigliotti J. C., Tin A., Pourafshar S. (2020). GSTM1 deletion exaggerates kidney injury in experimental mouse models and confers the protective effect of cruciferous vegetables in mice and humans. *Journal of American Society of Nephrology*.

[157] Le T. H. (2021). GSTM1 gene, diet, and kidney disease: implication for precision medicine?: recent advances in hypertension. *Hypertension*.

[158] Hosseinpour-Niazi S., Mirmiran P., Fallah-Ghohroudi A., Azizi F. (2015). Non-soya legume-based therapeutic lifestyle change diet reduces inflammatory status in diabetic patients: A randomised cross-over clinical trial. *British Journal of Nutrition*.

[159] Navarro S. L., Schwarz Y., Song X. (2014). Cruciferous vegetables have variable effects on biomarkers of systemic inflammation in a randomized controlled trial in healthy young adults. *Journal of Nutrition*.

[160] Hussain Y., Khan H., Alsharif K. F., Khan A. H., Aschner M., Saso L. (2022). The therapeutic potential of kaemferol and other naturally occurring polyphenols might be modulated by Nrf2-ARE signaling pathway: Current status and future direction. *Molecules*.

[161] Chen A. Y., Chen Y. C. (2013). A review of the dietary flavonoid, kaempferol on human health and cancer chemoprevention. *Food Chemistry*.

[162] Barve A., Chen C., Hebbar V., Desiderio J., Saw C. L. L., Kong A. N. (2009). Metabolism, oral bioavailability and pharmacokinetics of chemopreventive kaempferol in rats. *Biopharmaceutics & Drug Disposition*.

[163] Tzeng C. W., Yen F. L., Wu T. H. (2011). Enhancement of dissolution and antioxidant activity of kaempferol using a nanoparticle engineering process. *Journal of Agricultural and Food Chemistry*.

[164] Kumar A., Gupta G. K., Khedgikar V. (2012). In vivo efficacy studies of layer-by-layer nano-matrix bearing kaempferol for the conditions of osteoporosis: a study in ovariectomized rat model. *European Journal of Pharmaceutics and Biopharmaceutics*.

[165] Jin Y., Wen J., Garg S. (2013). Development of a novel niosomal system for oral delivery of *Ginkgo biloba* extract. *International Journal of Nanomedicine*.

[166] Sairazi N. S. M., Sirajudeen K. N. S. (2020). Natural products and their bioactive compounds: neuroprotective potentials against neurodegenerative diseases. *Evidence-Based Complementary and Alternative Medicine*.

[167] Kazmi I., Al-Abbasi F. A., Afzal M., Altayb H. N., Nadeem M. S., Gupta G. (2021). Formulation and evaluation of kaempferol loaded nanoparticles against experimentally induced hepatocellular carcinoma: in vitro and in vivo studies. *Pharmaceutics*.

[168] Safarpour S., Pirzadeh M., Ebrahimpour A. (2022). Protective effect of kaempferol and its nanoparticles on 5-fluorouracil-induced cardiotoxicity in rats. *BioMed Research International*.

